# Transpulmonary thermodilution: advantages and limits

**DOI:** 10.1186/s13054-017-1739-5

**Published:** 2017-06-19

**Authors:** Xavier Monnet, Jean-Louis Teboul

**Affiliations:** 1 Hôpitaux Universitaires Paris-Sud, Hôpital de Bicêtre, Medical Intensive Care Unit, Le Kremlin-Bicêtre, F-94270 France; 2 Université Paris-Sud, Faculté de médecine Paris-Sud, Inserm UMR S_999, Le Kremlin-Bicêtre, F-94270 France; 3 Service de réanimation médicale, Hôpital de Bicêtre, 78, rue du Général Leclerc, F-94270 Le Kremlin-Bicêtre, France

**Keywords:** Haemodynamic monitoring, Cardiac output, Extravascular lung water, Fluid responsiveness, Cardiac preload

## Abstract

**Background:**

For complex patients in the intensive care unit or in the operating room, many questions regarding their haemodynamic management cannot be answered with simple clinical examination. In particular, arterial pressure allows only a rough estimation of cardiac output. Transpulmonary thermodilution is a technique that provides a full haemodynamic assessment through cardiac output and other indices.

**Main body:**

Through the analysis of the thermodilution curve recorded at the tip of an arterial catheter after the injection of a cold bolus in the venous circulation, transpulmonary thermodilution intermittently measures cardiac output. This measure allows the calibration of pulse contour analysis. This provides continuous and real time monitoring of cardiac output, which is not possible with the pulmonary artery catheter. Transpulmonary thermodilution provides several variables beyond cardiac output. It estimates the end-diastolic volume of the four cardiac cavities, which is a marker of cardiac preload. It provides an estimation of the systolic function of the combined ventricles. It is more direct than the pulmonary artery catheter, but does not allow the distinct estimation of right and left cardiac function. It is easier and faster to perform than echocardiography, but does not provide a full evaluation of the cardiac structure and function. Transpulmonary thermodilution has the unique advantage of being able to estimate at the bedside extravascular lung water, which quantifies the volume of pulmonary oedema, and pulmonary vascular permeability, which quantifies the degree of a pulmonary capillary leak. Both indices are helpful for guiding fluid strategy, especially in case of acute respiratory distress syndrome.

**Conclusions:**

Transpulmonary thermodilution provides a full cardiovascular evaluation that allows one to answer many questions regarding haemodynamic management. It belongs to the category of “advanced” devices that are indicated for the most critically ill and/or complex patients.

## Background

**Fig. 1:**
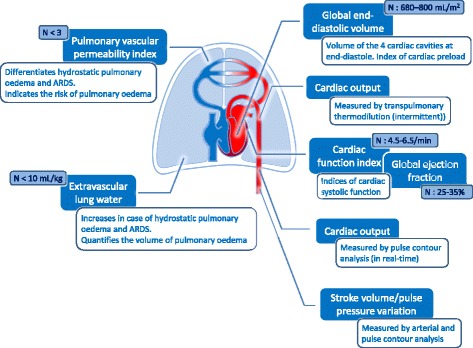
Haemodynamic variables provided by transpulmonary thermodilution and calibrated pulse contour analysis, with their meaning, utility and normal values (*N*). *ARDS* acute respiratory distress syndrome

A task force of the European Society of Intensive Care Medicine recently recommended using advanced haemodynamic monitoring in severe shock and complex situations and stated that the pulmonary artery catheter and transpulmonary thermodilution (TPTD) devices are suitable for this purpose [1]. The TPTD technique emerged in the early 2000s. The PiCCO (Pulsion Medical Systems, Munich, Germany) and the Volume View (Edwards LifeSciences, Irvine, United States of America) devices measure cardiac output but also provide several other valuable pieces of haemodynamic information (Fig. 1). In our opinion, the approach of TPTD is quite different from pulmonary artery catheterisation in many repects. The attractiveness of this approach and the fact that the technique is easy to set up likely explain why TPTD use has increased. Which methods are used by TPTD to measure cardiac output and the other variables and what can be said regarding the validation of these variables? What are the indications of TPTD and what is its place in the haemodynamic management of ICU and surgical patients? These are the questions we address in this review.

## Measurement of cardiac output

### How does TPTD measure cardiac output?

TPTD requires the injection of a bolus of cold saline in the superior vena cava territory. At the tip of a femoral arterial catheter (the tip stands in the iliac artery), a thermistor senses the decrease in blood temperature. As with standard pulmonary thermodilution, TPTD measures cardiac output by using the Stewart–Hamilton principle. Compared to the pulmonary artery catheter, the difference is that, with TPTD, cold saline is injected not in the right atrium but in a central vein and the blood temperature is measured not in the pulmonary artery but in a systemic artery.

### Is TPTD accurate for measuring cardiac output?

From all the studies that have investigated the validity of TPTD [2], it can be reasonably concluded that this measurement of cardiac output is accurate compared to the pulmonary artery catheter and the Fick method. Besides its accuracy, the ability of TPTD devices to reliably track changes in cardiac output is also important. It is recommended to average the result of three bolus injections [3]. In such a case, the smallest change in cardiac output that can be accurately detected by TPTD is 12% (this is the “least significant change” of the technique) [4]. It is comparable with the pulmonary artery catheter [5].

The reliability of the measurement requires a significant difference in blood temperature induced by TPTD. According to the manufacturer’s recommendations, 15 mL of saline <8 °C must be injected. Therapeutic hypothermia does not affect the precision of the measurement of cardiac output by TPTD [6]. It seems that boluses at room temperature create a sufficient difference [7], although this may result in a slight but significant overestimation of cardiac output [8]. Very importantly, great caution should be applied when performing thermodilution (volume of injection, absence of leaks in the circuit, regularity of the speed of injection, bolus temperature), since any error in the thermodilution curve may affect the estimation of cardiac output but also of all variables that are inferred from the curve analysis.

When the venous catheter is inserted not in the superior vena cava but in a femoral vein, contralateral to the arterial catheter, the measurement of cardiac output is unaffected, while it is not the case for thoracic volumes measured by the technique (see below) [9, 10]. Of course, the arterial catheter must not be inserted in the femoral site on the same side as the venous catheter used for injection. Injecting the bolus in a port-a-catheter provides reliable measurements of cardiac output and its variations [11]. The technique cannot be used in case of extracorporeal membrane oxygenation. By contrast, continuous veno-venous haemofiltration does not affect the reliability of cardiac output measurement by TPTD [12–14], even for high blood pump flows [13]. The thermistor-tipped catheter is most often inserted in the femoral artery, but the axillary, brachial and radial (long catheter) arteries may also be used, although this requires that the elbow remains in the extended position.

### What are the limitations of TPTD for measuring cardiac output?

One drawback of TPTD is that recirculation of the cold indicator is larger than with pulmonary thermodilution [2]. Also, if cardiac output is very low, typically below 2 L/min, TPTD devices do not provide any measure because of uncertainty about the technique’s reliability. The loss of injectate temperature is greater than with conventional thermodilution. In this regard, high volumes of lung water may theoretically amplify the loss of injectate temperature through the pulmonary circulation, even though this phenomenon is likely negligible [15]. Finally, the main drawback of TPTD for measuring cardiac output is that it performs only intermittent measurements (Table 1). It cannot detect short term changes, as induced by mechanical ventilation, passive leg raising [16] or end-expiratory occlusion [17] tests, for instance.

**Table 1:** Advantages and drawbacks of measurements performed by transpulmonary thermodilution and calibrated pulse contour analysis

Variable	Main advantages	Main drawbacks
Cardiac output measured by TPTD	As reliable as pulmonary thermodilution	Does not provide a continuous measurement
Cardiac output measured by pulse contour analysis	Continuous measurement Precise measurement Assesses short-term and small changes	Requires regular recalibration
Global end-diastolic volume	Better reflects cardiac preload than pressure markers of preload	Does not distinguish between the right and left ventricles Less directly reflects the risk of pulmonary oedema than PAOP
Stroke volume variation	Continuous automated assessment of fluid responsiveness	Cannot be used in case of spontaneous breathing, cardiac arrhythmias and ARDS
Cardiac function index, global ejection fraction	Can be used as an alarm for decreased LV systolic function	Overestimate LV systolic function in case of right ventricular dilation Indirect markers of cardiac systolic function Do not precisely assess cardiac structure and function
Extravascular lung water	Directly estimates the volume of lung oedema	Unreliable in case of pulmonary embolism, lung resection, large pleural effusions
Pulmonary vascular permeability index	Directly estimates lung permeability Distinguishes hydrostatic from permeability pulmonary oedema	Same as for extravascular lung water

*ARDS* acute respiratory distress syndrome, *LV* left ventricular, *PAOP* pulmonary artery occlusion pressure, *TPTD* transpulmonary thermodilution

### Calibration of pulse contour analysis

Besides TPTD, the PiCCO and VolumeView devices estimate cardiac output by analysis of the arterial curve (“pulse contour”) sampled through the arterial catheter. This allows real-time monitoring of cardiac output.

Pulse contour analysis is based on the relationship between stroke volume and the amplitude and shape of the aortic pressure curve [18]. Devices analyse the geometry of the pressure waveform recorded in a peripheral artery, estimate the arterial curve at the aortic level, and estimate stroke volume from the geometrical properties of the pressure waveform through proprietary algorithms.

The measure of cardiac output by pulse contour analysis is very precise, more so than with TPTD [5], but it may drift over time, especially when the arterial resistance changes [19–21]. Then, the PiCCO and Volume View devices calibrate pulse contour analysis by the value obtained by TPTD each time TPTD is performed. Compared to uncalibrated pulse contour analysis devices, calibrated ones have better accuracy, especially when arterial tone changes to a significant extent, for instance under vasopressors [20, 22]. Calibration of pulse contour analysis should be encouraged after a 1-h calibration-free period [23]. This does not mean that calibration must be performed every hour in patients with a TPTD device, but rather that, when cardiac output is mandatory for interpreting the haemodynamic condition, calibration of pulse contour analysis must be performed if 1 h or more has elapsed since the last calibration (Table 1).

## Assessment of cardiac preload: global end-diastolic volume

### What is the global end-diastolic volume?

**Fig. 2:**
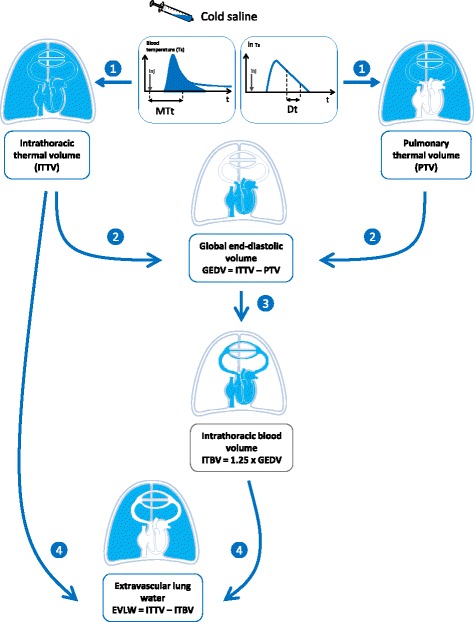
Assessment of intrathoracic volume by the PiCCO device. With the VolumeView device, the geometrical analysis of the thermodilution curve directly estimates the global end-diastolic volume. *Dt* downslope time, *MTt* mean transit time. For explanation, see the text

Besides cardiac output, TPTD estimates some intrathoracic volumes of great pathophysiological interest (Fig. 1). This estimation is based on the analysis of both the thermodilution curve and its logarithmic transformation (Fig. 2). According to the Stewart–Hamilton principle, the total distribution volume of the cold indicator between the injection and detection sites, the *intrathoracic thermal volume*, is obtained by multiplying cardiac output by the mean transit time of the cold indicator. According to the Newman principle, the largest distribution volume of the cold indicator between the injection and detection sites, the *total pulmonary volume*, is obtained by multiplying cardiac output by the downslope time of the thermodilution curve. Then, subtracting the *pulmonary thermal volume* from the *intrathoracic thermal volume* estimates the *global end-diastolic volume* (GEDV). This corresponds to the volume of all four cardiac chambers at the end of diastole (Fig. 2). Whereas the PiCCO device assesses GEDV according to the Newman principle, the VolumeView device assesses it from a different geometrical analysis of the thermodilution curve, which is based on the slopes of the up and down parts of the curve and a proprietary function [24]. Nevertheless, both techniques have been demonstrated to be interchangeable [25].

### Is GEDV valid as a measure?

GEDV behaves as it is typically expected from a marker of cardiac preload [26]. In patients with septic shock, GEDV increased with fluid administration but remained constant during dobutamine administration, despite similar increases of cardiac output [26]. It has been suspected that there is a mathematical coupling between GEDV and cardiac output since both variables are derived from the same thermodilution curve, but this has not been confirmed by clinical investigations [26–28].

GEDV includes the cardiac volume and also a part of the superior vena cava volume and the volume of the aorta that are between the bolus injection site and the thermistor. This may explain why GEDV is larger than the actual volume of the four cardiac chambers. Nevertheless, these vascular volumes are roughly unchanged during changes in cardiac preload. It has been demonstrated that GEDV reliably tracks the changes in volumetric preload induced by volume expansion when compared to echocardiographic measurements, better than the volumetric assessment allowed by some modified versions of the pulmonary artery catheter [29, 30].

The least significant change of GEDV when three cold boluses are injected is 12% [4]. If boluses are injected through the femoral vein, the normal values of GEDV are higher than if they are injected through the superior vena cava, since it includes the volume of the inferior vena cava [9, 10]. They can be estimated from formulas using a patient’s biometric data [10]. The changes in “femoral” GEDV are correlated with those of “superior vena cava” GEDV [10].

### Measures of cardiac preload volumes or pressures?

It has been argued that volume markers of cardiac preload (end-diastolic volume measured by TPTD or echography) are better than pressure markers (central venous pressure, pulmonary artery occlusion pressure (PAOP)) since studies reported that left ventricular end-diastolic dimensions were better correlated with stroke volume than PAOP [31–33].

Nevertheless, two points must be kept in mind. First, if the ventricular compliance is low, small changes in volume induce larger changes in pressure, and changes in volume may underestimate changes in cardiac preload. Second, the risk of hydrostatic pulmonary oedema, the formation of which directly depends on the hydrostatic pressure gradient between the pulmonary capillary and interstitium [34], is better indicated by PAOP than by any volume indices.

A specific limitation of GEDV is that it does not distinguish between left and right cardiac preload. In practice, in case of right ventricular dilation, GEDV is increased while the left ventricular preload is normal [35, 36].

### How do we use GEDV in practice?

Like all static markers of cardiac preload, GEDV is a poor indicator of fluid responsiveness [37]. Nevertheless, this does not mean that it is useless. Knowing the level of cardiac preload is essential for determining the shock origin when it is unclear. Also, during volume expansion, cardiac preload must be measured in order to check that it actually increases and that fluid has not just been diluted in the vasodilated venous reservoir [26].

## Cardiac systolic function

### Two indices provided by TPTD

TPTD estimates the left ventricular systolic function through the *cardiac function index* and the *global ejection fraction*. Cardiac function index is the ratio of cardiac output (measured by TPTD) and GEDV (Fig. 1). The global ejection fraction is equal to stroke volume divided by GEDV and multiplied by 4, assuming that the *left ventricular* end-diastolic volume is a quarter of GEDV. Of course, this is a rough assumption since all cardiac cavities are not of similar volume.

Some studies showed a good correlation between absolute values of cardiac function index and of the left ventricular ejection fraction (LVEF) measured by echocardiography in animals [38] and in patients [35, 36, 39]. Some specific cut-off values of cardiac function index and global ejection fraction accurately detected low values of LVEF [35, 36, 38, 40]. Moreover, changes in cardiac function index and global ejection fraction track changes in echocardiographic LVEF [35, 36, 40], especially when induced by inotropes [36, 40]. The least significant change of cardiac function index is 12% when three cold boluses are injected [4].

### What are the limitations?

As for GEDV, the main limitation of cardiac function index and global ejection fraction comes from the dilation of the right cardiac chambers. In this situation, the GEDV is increased and the cardiac function index and global ejection fraction are decreased while the left ventricular contractility is unchanged [35, 36, 38, 40]. Also, although this has not been formally investigated, cardiac function index and global ejection fraction likely share with LVEF the limitation that they depend on left ventricular preload and afterload [41]. Moreover, even though it is possible to identify threshold values in order to detect low LVEF, the correlation between LVEF and cardiac function index obviously cannot be perfect since the cardiac function index is a marker of the “global” cardiac systolic function [35, 36, 38, 40] (Table 1). Echocardiography remains the most accurate bedside technique for measuring LV systolic function. Finally, echocardiography has the advantage of performing a complete assessment of cardiac structure and function, while it is not allowed by TPTD (Table 1).

### What is the place of TPTD indices of systolic function in practice?

Echocardiography requires many more skills than TPTD. Moreover, it is rather tedious and time consuming, so it may not be performed as often as it should be during acute circulatory failure. Though the correlation between cardiac function index and LVEF is not perfect [35, 36, 39], every time thermodilution measurements are performed, global ejection fraction and cardiac function index give a rapid estimation of cardiac systolic function. They can easily warn clinicians that the left ventricular contractility is deteriorating and encourage them to perform echocardiography, which would not have been performed otherwise. In a patient that is receiving inotropes, TPTD systolic function indices also allow clinicians to follow the treatment effect [36].

### Extravascular lung water

Extravascular lung water (EVLW) is fluid that accumulates in the interstitial and alveolar spaces (Fig. 1). EVLW increases because of increased lung permeability or because of increased hydrostatic pressure in the pulmonary capillaries, or both [42]. Since it is the main pathophysiological pattern of hydrostatic pulmonary oedema and of acute respiratory distress syndrome (ARDS), it might be of very special help for establishing diagnosis, assessing the severity of the disease and guiding the therapeutic strategy.

### How does TPTD measure lung water?

With the PiCCO device, the estimation of EVLW is performed by subtracting the intrathoracic blood volume from the intrathoracic thermal volume (Fig. 2). The intrathoracic blood volume is estimated by multiplying GEDV by 1.25 [43]. The VolumeView device is based on the same principle but, as explained above, it estimates GEDV differently from the PiCCO device [24, 25] (Fig. 2). However, a good agreement between the values provided by both devices has been reported [24, 25].

The EVLW value is indexed to the predicted body weight, not the actual body weight, in order to avoid underestimation of EVLW [44]. When three cold boluses are used, the least significant change in EVLW is 12% [4]. Using room temperature boluses results in a slight but significant overestimation of EVLW [8].

### Is the estimation of EVLW valid?

Several arguments support the reliability of the estimation of EVLW by TPTD. First, it has been validated against transpulmonary thermo-dye dilution in humans [43], and against gravimetry, the reference technique, in animals [45, 46] and humans [47]. Second, experimental [48, 49] and clinical [4, 50] studies demonstrated that the TPTD estimation of EVLW is precise. In an autopsy study in 30 patients, the correlation coefficient between EVLW measured by TPTD and by gravimetry was 0.90 [47]. TPTD was able to detect the small and short-term changes in EVLW induced by bronchoalveolar lavage [50]. With TPTD, it is also possible to detect the rapid increase in EVLW that occurs during a weaning-induced pulmonary oedema, sometimes over a few minutes only [51]. Third, the reliability of the TPTD estimation of EVLW is indirectly supported by studies showing that EVLW predicts mortality in critically ill patients [52–56] independent of other severity indices [52–54], which would be impossible to establish if the TPTD measurement of EVLW was unreliable.

### What are the potential limitations of EVLW measurement?

In case of vascular occlusion due to pulmonary embolism, the volume of distribution of the cold indicator is reduced, resulting in underestimation of EVLW [57] (Table 1). Nevertheless, the occlusion of some small-diameter vessels during ARDS, which results from vascular remodelling, microthrombi, hypoxic vasoconstriction or positive end-expiratory pressure (PEEP), has no impact on the TPTD estimation of EVLW [58, 59].

The theoretical effects of PEEP on the measurement of EVLW by TPTD are contradictory. PEEP squeezes some pulmonary vessels and reduces the distribution volume of the cold indicator. By contrast, PEEP could recruit some atelectatic regions and alleviate the hypoxic constriction that prevents the thermal indicator from reaching such regions. By decreasing cardiac output, PEEP may also reduce the pulmonary microvascular hydrostatic pressure and hence EVLW. On the contrary, PEEP increases the central venous pressure, which may impede the lymphatic drainage of EVLW. Studies investigating the net effect of these mechanisms in clinical practice are scarce. One study reports that, in ARDS patients, there was a strong correlation between the EVLW measured by transpulmonary thermo-dye dilution and lung weight measured by computed tomography over a broad range of PEEP levels [60].

*Lung resection* logically decreases the volume of EVLW [61, 62] but TPTD may overestimate the remaining volume of EVLW [61]. Similarly, the estimation of EVLW by TPTD is significantly affected by one-lung ventilation [63].

*The type of ARDS* may affect the reliability of TPTD for estimating EVLW. In heterogeneous forms of ARDS, the pulmonary blood flow may be redistributed away from oedematous areas, which could lead to underestimation of EVLW [64], even though this redistribution phenomenon has been demonstrated to be severely blunted [65].

*Pleural effusions* of large volume could induce an overestimation of EVLW because the cold indicator also diffuses in the pleural liquid [66]. Nevertheless, a study found that, by contrast, removing pleural effusion increased EVLW, perhaps by alleviating atelectasis in contact with the pleural effusion [67]. As for cardiac output or GEDV, *renal replacement therapy* [12, 13] and therapeutic hypothermia [6] do not alter the measurement of EVLW.

### Pulmonary vascular permeability index

TPTD estimates the permeability of the alveolo-capillary barrier through the pulmonary vascular permeability index, which is the ratio of EVLW over pulmonary blood volume, i.e. the ratio of the fluid volume that is out of the vessels over the fluid volume that remains in the vessels [68–70].

This index has been validated in animal [45] and clinical [68–70] studies showing that it is lower in hydrostatic pulmonary oedema than in ARDS. A pulmonary vascular permeability index value of 3 was found to be the best threshold for distinguishing between both forms of pulmonary oedema [68, 69] and should be considered as the maximal normal value. The number of human studies on the index is small for the moment, so that the normal value might be slightly different from 3. The pulmonary vascular permeability index shares the same limitations as EVLW.

### How do we use EVLW and the pulmonary vascular permeability index in clinical practice?

#### Definition of ARDS

Although an increase in lung water and permeability is the pathophysiological hallmark of ARDS, its current definition does not take them into account [71]. In order to exclude hydrostatic pulmonary oedema, the Berlin definition only stipulates that the filling pressure of the left ventricle should not be elevated. Not only is this obviously a very indirect estimation of lung permeability, but also an elevated filling pressure of the left ventricle does not preclude that lung permeability is increased, especially after a few days of fluid resuscitation. Moreover, some clinical studies suggest that taking EVLW and/or pulmonary vascular permeability index values into account has helped to predict the progression to lung injury in patients with risk factors 2.6 ± 0.3 days before the patients met American-European Consensus Conference criteria of ARDS [72]. Changes in the pulmonary vascular permeability index should thus be taken into consideration for following worsening or improvement of ARDS in clinical practice. In another study, the value of EVLW was in close relationship with the severity of ARDS as defined by the categories of the Berlin definition [73]. It has also been shown that the use of EVLW improves by up to eightfold the post-test odds ratio for the diagnosis of acute lung injury, ARDS and severe lung injury [70]. At the least, all these reasons argue for using TPTD in order to better characterise the ARDS pattern [74, 75]. Of course, the cost of such devices prevents their use as a standard for every ARDS patient across the world.

#### Fluid management

In order to avoid fluid overload in critically ill patients, fluids should be administered only if preload responsiveness has been assessed by appropriate indices [76]. In addition, the risk of fluid therapy should also be considered. EVLW indicates the volume of water that has already leaked toward the lung interstitium and alveoli, while the pulmonary vascular permeability index indicates a priori the risk of leakage. In patients with ARDS, if EVLW and the pulmonary vascular permeability index are much higher than their normal values, fluid administration should be as restricted as possible.

In the context of ARDS, it has been reported that management based on protocols including EVLW measurements is safe [77], leads to a lower cumulative fluid balance [78], decreases ICU mortality [77], and reduces the duration of mechanical ventilation [78] and of ICU stay [78]. Some studies have provided different results. In a mixed population of ICU patients, TPTD was associated with an increased fluid balance [79]. In another study, TPTD-based fluid management did not improve outcome when compared to central venous pressure-based fluid [80]. Nevertheless, the protocols associated with the two latter studies have been strongly criticised [81, 82]. In studies investigating monitoring devices, the effect on prognosis is tightly related to the quality of the protocol attached to the device. Whatever the device, results from questionable management protocols are inherently questionable [81].

#### Weaning from mechanical ventilation

Our group demonstrated that an increase in EVLW during a spontaneous breathing trial was able to diagnose weaning-induced pulmonary oedema with good accuracy, in particular with 100% specificity [51]. This does not mean that a TPTD device should be set up only for the purpose of detecting weaning-induced pulmonary oedema, but rather that, if the device is already in place, one should pay attention to EVLW during weaning trials.

## Side effects of TPTD

TPTD is an invasive technique although the invasiveness is not very different from the pulmonary artery catheter, even though it is easier to set up. Nevertheless, in a multicentre prospective series of 514 patients, the most common complications were small local haematomas after insertion (4.5%) and removal (1.2%) of the catheter. Other complications such as ischaemia (0.4%), pulse loss (0.4%) or femoral artery thrombosis (0.2%) were uncommon and transient, and all resolved with catheter removal or embolectomy [83]. This study is the only one that investigated such complications, and these results should be taken with caution. Nevertheless, in our opinion, they suggest that the technique has acceptable rates of complications when compared to the other risks incurred by critically ill patients.

The technique is contraindicated in case of femoral vascular prostheses. In our practice, in patients with arteriopathy, if two attempts to insert the catheter and the guide fail, we abandon the option of using the technique in this patient without further attempts. The weight of these complications must be compared to the severity of the patient condition, unacceptable for monitoring surgical interventions of patients at low risk but justified for high-risk surgical patients or critically ill patients [84].

## The place of TPTD among haemodynamic monitoring devices

### Haemodynamic monitoring: for which patients?

#### In the peri-operative setting

In the peri-operative setting, haemodynamic monitoring should be used to detect hypovolemia or low oxygen delivery for early prevention. In spite of its invasiveness, advanced haemodynamic monitoring should be preferred over less invasive devices in two circumstances. The first is when the patient is particularly complex and when more variables than just cardiac output are mandatory, such as during cardiac surgery and prolonged major surgery for instance [84]. The second is when less invasive techniques are more likely to be unreliable, as for instance uncalibrated pulse contour analysis during liver surgery because major changes in vasomotor tone are expected [19]. Several studies conducted in cardiac surgery and major abdominal surgery have shown that cardiac output monitoring led to a reduction in the rate of complications and the length of stay [85].

#### In the intensive care unit

In contrast to the peri-operative setting, no study has compared management with versus without haemodynamic monitoring in terms of mortality. In this context, demonstrating such a benefit would be very difficult. The prognosis of critically ill patients is influenced by so many factors that it is hard to believe a monitoring device alone could influence mortality. Many other monitoring techniques are used in ICU patients, such as electrocardiogram or blood gas analysis, while they have never been shown to improve prognosis. The decision to use haemodynamic monitoring in severe ICU patients should not be guided by outcome studies but by studies showing that it provides more complete information than basic monitoring with heart rhythm and blood pressure. In this regard, changes in arterial pressure only roughly detect changes in cardiac output [86, 87], especially when vasopressors change the arterial tone [86], monitoring EVLW can lead to decreases in fluid balance and advanced monitoring may change clinical decisions [88].

**Fig. 3:**
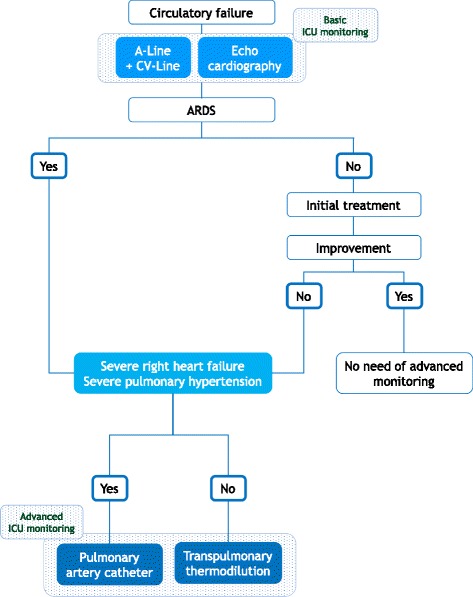
Indication for haemodynamic monitoring in the intensive care unit. *A-line* arterial line, *CV-line* central venous line. The choice also depends on team experience

Based on such arguments, recent recommendations state that haemodynamic monitoring is mandatory in patients with hypotension that resists initial fluid therapy [1] (Fig. 3). Advanced monitoring is particularly warranted in patients with ARDS and in patients where the dose of vasopressor is high and/or increasing (Fig. 3). Nevertheless, it seems that this is less common in practice [89] than recommended by a recent consensus [1].

### Which TPTD device?

Today, two TPTD devices are commercially available: the PiCCO system, which is integrated in the ProAQT platform (Pulsion Medical Systems, Munich, Germany), and the VolumeView system, which is incorporated in the EV1000 platform (Edwards Life Sciences, Irvine, USA). Of course, the manufacturers’ software packages are proprietary and not open, but both systems work on the same principles and technological differences between them are likely very small. In two studies, the measurements of cardiac output and volumetric variables were similar between both devices [24, 25]. In one study, pulse contour analysis by the VolumeView method was found to have a slightly better precision than by the PiCCO system [90]. The VolumeView device overestimates GEDV and the derived variables when the inferior vena cava route is used for injecting the cold bolus, and corrections that have been described for the PiCCO reduce this overestimation [91].

### TPTD versus pulmonary artery catheter?

TPTD/calibrated pulse contour analysis and traditional pulmonary thermodilution are both sophisticated techniques that provide several haemodynamic variables besides cardiac output. In this regard, both are suitable for complex and critically ill patients [1, 3, 84, 92]. An advantage of traditional pulmonary thermodilution is that it does not require recalibration. With the pulmonary artery catheter, however, semi-continuous estimation of cardiac output is not measured in real time and the response to a change in cardiac output is delayed by several minutes [93]. With calibrated pulse contour analysis, the displayed value of cardiac output is an average calculated over a few seconds only. Besides cardiac output, both techniques do not provide the same variables. PAOP has been repeatedly demonstrated to be unreliable for predicting fluid responsiveness, while calibrated pulse contour analysis allows one to perform passive leg raising or end-expiratory occlusion tests [76].

In order to decide to stop fluid administration, a given value of PAOP may correspond to different risk levels of increasing EVLW depending on the level of lung permeability [42]. For instance, fluid infusion at a PAOP of 12 mmHg would induce absolutely no lung oedema in a patient with normal lungs, while it would be extremely prone to increase EVLW in a patient with severe ARDS. Regarding the systolic function, in our opinion, the cardiac function index and global ejection fraction provide a more direct estimation than TPTD.

Unlike TPTD devices, the pulmonary artery catheter provides a direct estimation of pulmonary artery resistance. Right heart failure and severe pulmonary hypertension represent specific indications for the pulmonary artery catheter (Fig. 3) [1, 92]. Nevertheless, errors in the measurement of cardiac output resulting from tricuspid regurgitations must be kept in mind, even though it seems to exist mostly for severe regurgitations [94]. Also, the pulmonary artery catheter measures both central venous pressure and pulmonary artery pressure, allowing the estimation of right and left cardiac functions, while TPTD only estimates the global cardiac function. Another strong advantage of the pulmonary artery catheter is that it directly measures oxygen saturation of mixed venous blood, not central venous blood.

In conclusion, both types of devices can be used to monitor the haemodynamic status of critically ill patients with shock, although they provide clinicians with different answers to their questions. Clinicians should choose the device they know the best. The pulmonary artery catheter has a specific indication in case of acute right heart failure and acute pulmonary hypertension [1] (Fig. 3).

### TPTD or echocardiography? No choice: TPTD and echocardiography!

In our opinion, clinicians do not need to choose between TPTD devices and echocardiography. Echocardiography is unique for a complete assessment of cardiac structure and function. It must be performed early in every patient with acute circulatory failure [1, 3] (Fig. 3). Nevertheless, it is difficult to consider echocardiography as a device for haemodynamic monitoring because of the time that is required for the ultrasound examination [95]. An advantage of TPTD over echocardiography is that nurse-driven approaches can be used and that several patients can be evaluated simultaneously. In addition, TPTD provides clinicians with information that is not offered by echocardiography, like EVLW. Our practice for monitoring the most critically ill patients is a combination of TPTD for continuous and repeated haemodynamic monitoring and echocardiography for punctual assessments of cardiac function (Fig. 3). In addition, TPTD measurements may trigger recourse to echocardiography if global ejection fraction or cardiac function index suddenly decrease.

## Conclusions

Beyond cardiac output, TPTD provides several indices that help answer questions that clinicians ask themselves during haemodynamic management. In particular, it is a unique tool for guiding fluid therapy because it estimates lung water and permeability. Its place is in the management of the most critically ill and/or complex patients, which requires a reliable, precise and global vision of the cardiopulmonary condition. It will be very interesting to see how progress in technology in the era of digital health will transform and improve TPTD [96].

## Abbreviations

ARDS: Acute respiratory distress syndrome
EVLW: Extravascular lung water
GEDV: Global end-diastolic volume
LVEF: Left ventricular ejection fraction
PAOP: Pulmonary artery occlusion pressure
PEEP: Positive end-expiratory pressure
TPTD: Transpulmonary thermodilution.
